# Topography and behavioral relevance of the global signal in the human brain

**DOI:** 10.1038/s41598-019-50750-8

**Published:** 2019-10-03

**Authors:** Jingwei Li, Taylor Bolt, Danilo Bzdok, Jason S. Nomi, B. T. Thomas Yeo, R. Nathan Spreng, Lucina Q. Uddin

**Affiliations:** 1 ECE, CIRC, N.1 & MNP, National University of Singapore, Singapore, Singapore; 2 Data Science Division, Gallup, Atlanta, GA USA; 3 Department of Psychiatry, Psychotherapy and Psychosomatics, Aachen University, Aachen, Germany; 4 JARA, Translational Brain Medicine, Aachen, Germany; 5 Parietal Team, INRIA, Neurospin, bat 145, CEA Saclay, 91191 Gif-sur-Yvette, France; 6 Department of Psychology, University of Miami, Coral Gables, FL USA; 7 Laboratory of Brain and Cognition, Montreal Neurological Institute, Department of Neurology and Neurosurgery, McGill University, Montreal, QC Canada; 8 Departments of Psychiatry and Psychology, McGill University, Montreal, QC Canada; 9 Neuroscience Program, University of Miami Miller School of Medicine, Miami, FL USA

**Keywords:** Cognitive neuroscience, Neuro-vascular interactions

## Abstract

The global signal in resting-state functional MRI data is considered to be dominated by physiological noise and artifacts, yet a growing literature suggests that it also carries information about widespread neural activity. The biological relevance of the global signal remains poorly understood. Applying principal component analysis to a large neuroimaging dataset, we found that individual variation in global signal topography recapitulates well-established patterns of large-scale functional brain networks. Using canonical correlation analysis, we delineated relationships between individual differences in global signal topography and a battery of phenotypes. The first canonical variate of the global signal, resembling the frontoparietal control network, was significantly related to an axis of positive and negative life outcomes and psychological function. These results suggest that the global signal contains a rich source of information related to trait-level cognition and behavior. This work has significant implications for the contentious debate over artifact removal practices in neuroimaging.

## Introduction

In the imaging neurosciences, the global signal (GS) is defined as the timeseries of signal intensity averaged across all voxels in the brain, gray matter, or cortical gray matter. It is well known that non-neuronal sources including physiological noise caused by respiratory and cardiac events^1,2^ and participant motion^3^ contribute to the GS. As a consequence, GS regression became a pervasively adopted step in processing of resting-state fMRI data to attenuate these and other sources of noise^4,5^. However, in addition to containing artifactual information from various sources, the GS also contains information about ongoing neural activity^6^. Combined fMRI-electrophysiological studies in macaque monkeys permit analysis of spatiotemporal covariation between neural signal fluctuations measured with implanted electrodes and concurrent hemodynamic signals measured with fMRI. In one study, spontaneous fluctuations in local field potentials exhibited widespread positive correlations with fMRI blood oxygen level dependent (BOLD) changes over the entire macaque cortex^7^. More recently, neural origins of the global signal were indicated by inactivation of a neuromodulatory region of the basal forebrain, the nucleus basalis of Meynert. The nucleus basalis gives rise to the principal cholinergic as well as GABAergic projections to the cortex. Reversible pharmacological inactivation of the nucleus basalis in macaques resulted in regionally specific suppression of the global signal ipsilateral to the injection, further demonstrating a direct neuronal source of the global signal^8^.

Total estimates of baseline neuronal processing also come from magnetic resonance spectroscopy studies using ^13^C radiotracers, which permit simultaneous measures of energy demand (CMR_O2_) in neurons and glia as well as neuronal activity as reflected by presynaptic release of the neurotransmitters glutamate and GABA^9^. Such work in rodents suggests that around 80% of neuronal energy in the cerebral cortex supports global neuronal activity at rest^10^. In light of these findings, Hyder and colleagues suggest that neither total baseline neuronal activity nor fluctuations in baseline neuronal activity can be neglected as merely representing non-neuronal factors^9^. This contention is in line with positron emission tomography fluorodeoxyglucose studies in humans demonstrating that global signal amplitude is linked to changes in baseline glucose metabolism^11^. Additional evidence for neuronal contributions to the GS comes from EEG studies. The amplitude of the GS has been shown to relate to vigilance^12^ and arousal^13,14^. Taken together, the electrophysiological, metabolic, and neuroimaging evidence clearly demonstrate that at least some neural information is carried in the GS.

A previous study demonstrated that GS correlations with each brain voxel follow a specific topography, with significant weights in the occipital lobe^3^. This spatial pattern is thought to reflect respiratory patterns, and has been exploited as a feature for artifact removal^15^. Other work has noted that GS correlations with each brain voxel are stronger in some functional brain networks than others, and they fluctuate in magnitude over time, as well as vary between individuals^16^. GS variability is higher in schizophrenia, and correlated with behavioral symptoms^17^. Differences in GS topographical representation have been documented between schizophrenia patients and controls^18^. Overall, while these previous findings hint at possible relationships between the GS and individual brain functional network architectures^16–18^, none of these studies have provided evidence of *spatial* structure in the inter-subject variability of global signal topography in healthy individuals.

A critical open question to address concerns how GS topography is related to human cognition and behavior. Here we systematically explored individual variation in GS topography and its relationship with individual differences on a range of cognitive and behavioral measures using canonical correlation analysis (CCA). We anticipated the existence of robust relationships between GS topography and phenotypic information, indicating functional relevance of this signal that is often discarded as noise.

## Results

### Descriptive Analyses: The GS manifests in a topographically specific manner

**Figure 1:**
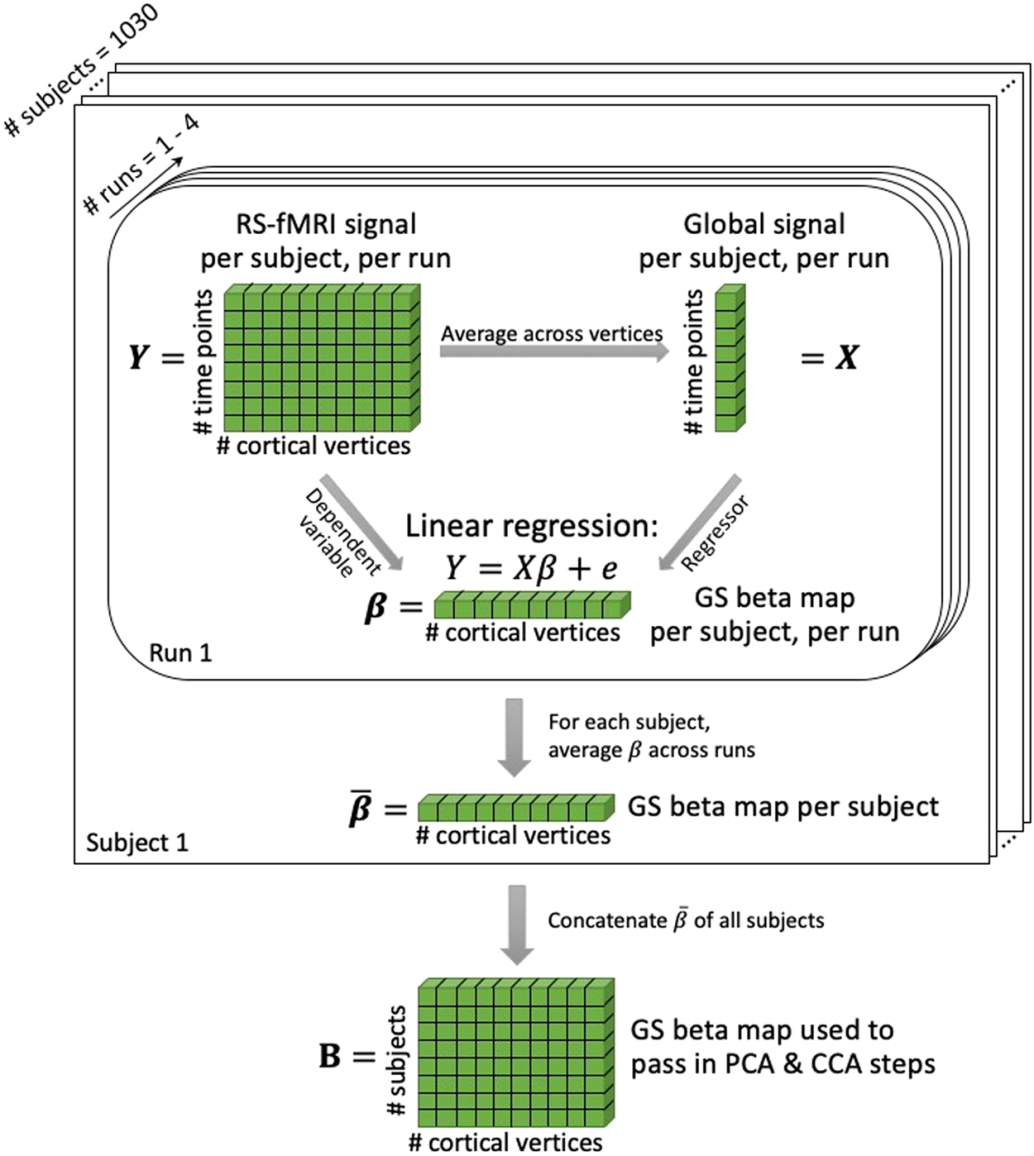
Illustration of global signal beta map calculation. For each subject and each run, the global signal (*X*) was computed as the averaged timeseries across all cortical vertices. The global signal was then regressed from the timeseries of each vertex (*Y*), resulting in the GS beta map of a given run (β). Averaging across all runs within a given subject, we obtained the GS beta map of that subject. The GS beta maps for each subject were then used in subsequent PCA and CCA analyses (**B**).

**Figure 2:**
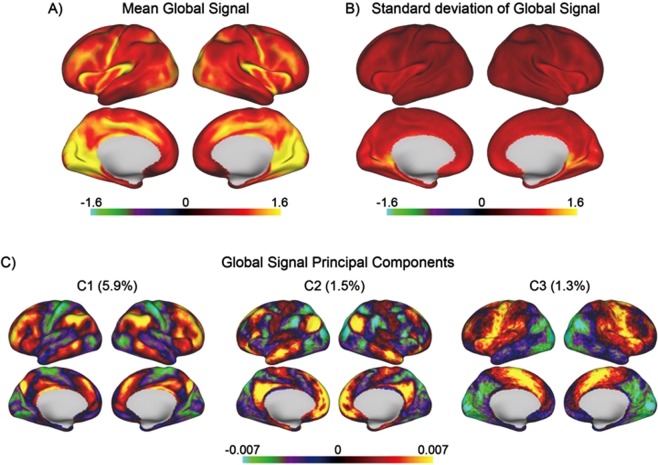
Global signal topography. (**A)** Brain regions dominating the global signal, computed as the mean global signal beta map across all subjects per vertex. Brain regions with strong global signal include the visual cortex, posterior insula, central sulcus and cingulate sulcus. (**B)** Brain regions with high individual variation (standard deviation) of global signal topography include retrosplenial and visual cortex. (**C)** Global signal principal components computed across subjects. The patterns resemble the canonical brain networks regularly observed from decompositions of resting-state fMRI data.

Capitalizing on the extensive neuroimaging and phenotypic de-identified data repository, the Human Connectome Project (HCP), we first assessed whether the GS has a distinctive spatial topography. Here, GS was operationalized by averaging the timeseries (t = 4800 time points from 4 scans for the majority of participants) across all surface-based cortical vertices for each participant. A GS beta map was then computed by regressing the GS at each vertex of each run of each subject (Fig. 1). The GS beta maps were then averaged across runs and across subjects yielding a mean GS beta map (Fig. 2A). The average whole-cortex GS beta map exhibited similar patterns to previous studies^3,18^, with strong mapping in the medial posterior occipital lobes, posterior insula, and central sulcus.

Examination of the mean global signal beta map may obscure potential independent patterns of GS expression that exist across subjects. For this reason, we also examined the standard deviation of the GS beta maps across subjects, with highest variance observed in retrosplenial and visual cortex (Fig. 2B). Next, we submitted all subjects’ GS beta maps to principal component analysis (PCA). The first three principal components, ranked by maximal variance explained, exhibited patterns of weights that resemble canonical functional networks: frontoparietal control network^19,20^, default^21^ and dorsal attention networks^22^, and sensorimotor and visual networks^23^ (Fig. 2C; Supplementary Fig. 1).

### Testing for brain-behavior correspondence of the GS: Canonical correlation analysis

**Figure 3:**
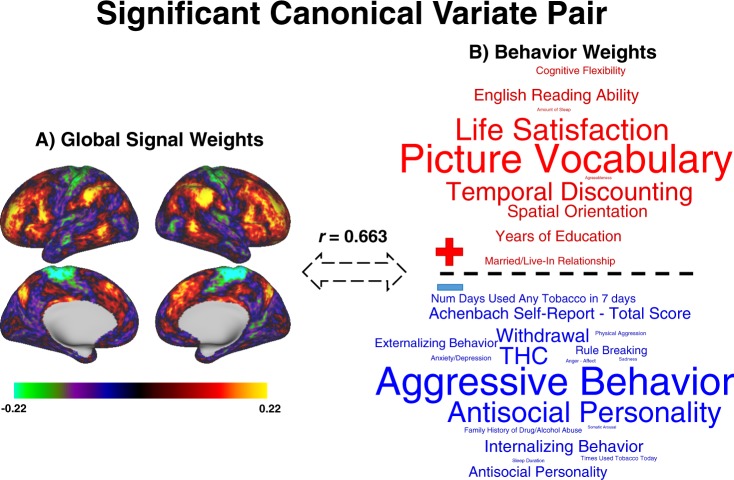
Individual differences in behavior associated with the global signal. (**A**) CCA weights of each vertex on the first canonical variate pair. Strong positive weights are observed in the frontoparietal and salience networks, and strong negative weights are observed in the motor cortex. (**B**) Top 20% positive (blue) and negative (red) behavioral variable CCA weights displayed in a word cloud. The size of the text in the word cloud is proportional to the absolute value of that variable’s CCA weight.

To examine potential relationships between whole-brain GS patterns and subject-level behavioral and cognitive measures, we conducted a CCA between the 100 principal components derived from the GS topography data describing regional GS contributions for each subject, and 100 principal components derived from the HCP behavioral data. Non-parametric permutation analysis for null-hypothesis testing revealed a single statistically significant population correspondence between GS topography and behavioral profiles (*r* = 0.667, *p* < 0.001; Fig. 3). The pattern of canonical modes for the GS closely followed spatial patterns that recapitulate the frontoparietal control network (positive weights) and sensorimotor and visual networks (negative weights). In assessing the similarity between the first principal component (Fig. 2C) and the first canonical variate (Fig. 3B), a vertex-to-vertex correlation showed high convergence in topography (*r* = 0.81, *p* < 0.001). The pattern of canonical component weights for the cognitive and behavioral subject measures followed a general positive/negative gradient of life outcomes and psychological function, similar to that observed in a previous CCA of interregional resting-state functional connectivity and behavior in the HCP dataset^24^.

### Robustness Analysis: Global signal topography and participant motion

**Figure 4:**
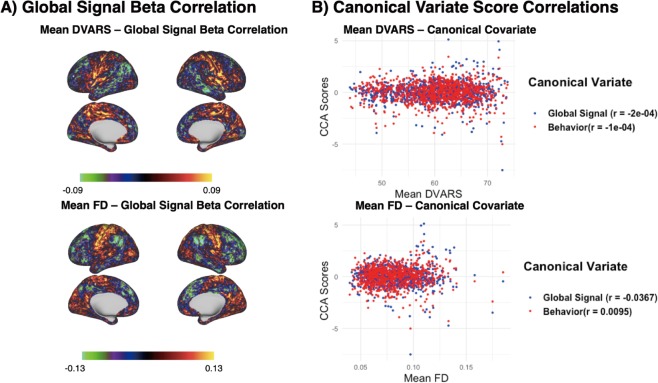
Correlation of Global Signal Estimates with Head Motion. (**A**) Per-vertex correlation between global signal beta estimates and average head motion (FD and DVARS) across subjects. (**B**) Scatter plots between global signal (blue) and behavioral (red) CCA scores and average head motion (FD and DVARS). Estimated Pearson correlation coefficients (*r*) are displayed in the legend beside each label.

Removal of the GS using linear regression has been proposed as the preferred method to attenuate participant motion related confounds in resting-state fMRI data^3,5^. Further, motion has been found to relate to individual differences in behavioral indices, suggesting that the propensity to move in the scanner (e.g. low task compliance), is a trait^25^. To confirm that the relationship between the GS and behavior we observed was not due to participant motion, we ran two more analyses: (1) computing the across-subject correlation between global signal beta estimates and mean head motion measures (DVARS, Framewise Displacement, FD) at each vertex, and (2) computing the across-subject correlation between canonical variate scores and mean head motion measures. As illustrated in Fig. 4, the brain topography associated with head motion is distinct from that observed to be associated with the positive/negative axis of behavior reported here (Fig. 3). In addition, no associations were observed between head motion (DVARS and FD) and the GS (*r* = −0.0002 and *r* = −0.0367, respectively) or behavior (*r* = −0.0001 and *r* = 0.0095, respectively). These supplementary analyses corroborate that meaningful associations between the GS and behavior are not likely to be mediated by head motion.

## Discussion

Resting-state fMRI has been widely embraced as a method to examine intrinsic functional brain networks^26^. Yet, researchers have acknowledged the challenges of separating neuronal and artifactual contributions in resting-state fMRI data^27^. The global signal (GS), or average signal intensity across the brain, is often removed from the timeseries via signal deconfounding to attenuate physiological and motion-related sources of noise^3,5^. However, the use of global normalization in fMRI data has long been debated^28^. Despite efforts towards consensus building with regards to the pre-processing step of removing the GS from fMRI timeseries via linear regression (GS regression)^29^, none has yet been attained^30,31^.

Studies measuring electrophysiology and brain metabolism provide ample indication for some neuronal component in the GS^7–9^. In addition, fMRI work demonstrates that any regressors unrelated to true data noise, for example, also remove variance with network structure^32^. As such, implementing data processing procedures to remove the GS from a given fMRI timeseries may inadvertently discard relevant structured neural signal, particularly if the dimensionality of the data is low^33^. To date, there have been few systematic investigations into the relevance of the GS for studies attempting to link brain connectivity and individual differences in behavioral phenotypes^34^.

The present investigation examines individual differences in the spatial topography of the GS, followed by a CCA of these brain-wide GS patterns with the rich repertoire of behavioral data provided by the Human Connectome Project. First, we found that the subject-wise overall GS was related to cortical grey matter in a topographically-specific manner. The first principal component of the global signal, explaining ~6% of the variance, was reminiscent of the canonical frontoparietal control network^19,20^ (positive weights) and sensorimotor and visual network (negative weights). The second component, explaining ~2% of the variance, represented the canonical anticorrelation between the default and dorsal attention networks (See Supplementary Fig. 1 for the first ten components of the GS).

Next, we conducted CCA using the principal components of the GS on the one hand, and phenotypic data on the other. This GS-behavior decomposition estimated using CCA aligns with a positive/negative axis of life experience and psychological indices. Greater frontoparietal control network weighting within the GS was significantly related to elevated scores on picture vocabulary, temporal discounting, life satisfaction and other measures on the positive axis, and lower aggressive antisocial behavior on the negative axis (Fig. 3). In fact, this positive/negative axis of behavior is remarkably similar to that previously observed, with a critical difference that we used the subject-wise GS beta maps rather than inter-regional connectivity matrices of resting-state fMRI data in the CCA^24^. There appear to be many similarities between the current results and those of Smith and colleagues in the brain regions associated with this positive-negative axis of behavior. Key differences to note are brain functional connections robustly involving the cingulum and anterior temporal lobes^24^. In contrast, we found the strongest behavioral associations with lateral prefrontal cortical areas, anterior inferior parietal lobule, and other regions which comprise the frontoparietal control network^19^. The current results lead to the surprising observation that significant brain-behavior relationships can be derived from GS beta maps alone.

A possible explanation for our findings could be the underlying relationship between GS beta maps and functional connectivity. A vertex’s value in the GS beta map represents how similar that vertex’s timeseries is to the averaged timeseries from the whole brain. Mathematically, this GS beta value relates to the mean functional connectivity between the given vertex and all vertices, including itself. Given the results from Smith and colleagues, one might predict that the mean functional connectivity of each vertex is associated with the behavioral measures. However, it is still meaningful to show that individual differences in GS topography contain sufficient neural information to be associated with individual variation in behaviors. This suggests meaningful differences in functional MRI sources of variance (GS topography versus connectivity) that are associated with behavior.

The present study extends beyond the work of Smith and colleagues in multiple ways. First, we show similar brain-behavior associations with positive/negative axes as that reported by Smith and colleagues, although we have exclusively focused on the global signal aggregates that are commonly treated as a nuisance source. So, while that study examined what is commonly considered to be BOLD signal of interest, our analyses centered on what is often removed from the BOLD signal before performing any target analyses. We have determined how the global signal is differentially weighted across cortex (eg. global signal topography), with distinct magnitudes and variance. Most previous work that incorporates the global signal in the analysis uses one value per brain scan, which ignores the topographic specificity of this aggregate statistic. It is important to note that this regional weighting may impact the residual signal of interest when GS regression is applied.

The current findings contribute to two substantive research areas in contemporary network neuroscience, one theoretical and one methodological. First, our results speak to the theoretical systems neuroscience question of what aspects of neural activity are critically implicated in individual differences in cognition and behavior. Intrinsic functional connectivity MRI is a well validated approach to delineate and characterize functional-anatomic brain networks^35,36^. This method is also capable of revealing important dynamic aspects of neural processing that play an active role in cognition^37,38^. Intrinsic functional connectivity is thought to index stable individual features^39^, and has been associated with individual differences in a number of behavioral and cognitive domains as well as overall intelligence^40,41^. Such work has emphasized how interregional synchrony of brain activity involved in cognitive operations is associated with individual differences in performance of these cognitive operations^42^. The current work provides a novel complement to prior observations, suggesting that the GS topography alone carries structured information related to large scale brain networks and accounts for variability in behavior across individuals.

Methodologically, the current findings are relevant for the question of what effect specific pre-processing strategies have on the outcome of a given functional connectomics analysis. The practice of GS regression has come under scrutiny for a number of reasons. Even as early as PET and fMRI work from 1998, the validity of adjusting for effects of GS changes was called into question, as it may meaningfully alter results and thus interpretation of studies^28^. In resting-state fMRI analyses, the practice of GS regression can have additional unintended consequences. After GS regression, correlation values are mathematically centered on zero, which can produce spurious negative correlation values^43^. Researchers have noted that differences in caffeine intake can affect the GS such that caffeine leads to widespread decreases in connectivity and global signal amplitude, suggesting a neuronal source for GS that varies across individuals and time^44^. Another concern has been highlighted with simulated fMRI data, in which GS regression has been shown to artificially introduce correlations between brain regions and distort group differences in inter-regional correlations^33^. A study of autism spectrum disorder demonstrated that GS regression leads to a reversal in the direction of group correlation differences relative to other preprocessing approaches, with a higher incidence of both long-range and local connectivity differences that favor the ASD group^45^. Although GS regression has been shown to mitigate the effects of several sources of noise on estimates of functional connectivity^3,5,46^, the findings highlighted above suggest that functional connectivity values derived after GS regression has been performed may need to be interpreted with great caution.

One reason that deconfounding via GS regression persists despite these concerns is that the procedure minimizes the relationship between functional connectivity and motion^47^. Head motion is clearly related to some aspects of the GS, as we again demonstrate in the current work (Fig. 4). Data denoising and the process of dealing with motion artifacts is particularly challenging when working with large samples of “legacy” data of varying quality^35^. The practice of treating the GS as unwanted variation of no scientific value unfortunately remains in effect.

A recent protocol for mitigating head motion artifact in functional connectivity MRI suggests that GS regression is “singular in its ability to remove widespread artifact^5^”. Meanwhile, GS regression has been shown to strengthen the association of functional connectivity data with multiple behavioral phenotypes across cognition, personality and emotion^34^, possibly because the gain of having cleaner data outweighs the loss of neural information in the GS for some phenotypes. Therefore, the current findings demonstrating a relationship between GS topography and behavioral phenotypes are not contradictory to the strengthened associations reported by Li and colleagues. Depending on different research questions^29^, researchers should take great care in the choice of removing the GS during data preprocessing.

Some leaders in the area of fMRI data analysis have suggested that “…the field has reached a consensus that, as a pre-processing step, the global signal should not be removed^48^”. Many agree that it will be critical to continue to probe and better understand sources that contribute to the GS in future work^6,30^. There is growing evidence that prospective data acquisition procedures such as multi-echo fMRI can provide a means to effectively remove motion artifacts in fMRI data^49–51^. The current results do not speak to the question of whether or not GS regression should be utilized as a data processing strategy. Instead, we illustrate what tradeoffs can be anticipated when this step is included. Our results highlight the fact that a specific signal cannot always be unambiguously categorized as a confounder or not^52^. The nature of artifactual influences in the domain of imaging neuroscience may take a different form as a consequence of data richness, with hundreds of phenotypic variables often recorded for each individual. Our findings suggest that the neuroimaging community may have to carefully reconsider current deconfounding practices. Ultimately, the GS in human neuroimaging appears to be both signal and noise.

## Methods

### Dataset

Our population neuroscience study utilized the Human Connectome Project (HCP) S1200 release^53^. Participants (N = 1094) were healthy young adults (ages 22–37) drawn from a population of twins and siblings. All imaging data were acquired on a customized Siemens 3 T Skyra at Washington University in St. Louis using a multi-band sequence. The structural images were 0.7 mm isotropic. The resting-state fMRI data were 2 mm isotropic with TR = 0.72 s. Two sessions of rs-fMRI data were collected on consecutive days for each subject, and each session consisted of one or two runs. The length of each rs-fMRI scan was 14.4 min (1200 frames). Details of the data collection can be found elsewhere^53,54^. Informed consent was obtained from all subjects. Details about behavioral measures can be found in HCP S1200 Data Dictionary and^55^. All methods were carried out in accordance with relevant guidelines and the University of Miami Institutional Review Board approved the study.

### Preprocessing of resting-state fMRI data

Preprocessing details of HCP data can be found elsewhere (HCP S1200 manual)^53,54,56^. ICA-FIX^57,58^ was applied for denoising. The surface (fs_LR) data were aligned with MSM-All^59^.

Motion censoring was performed to remove subjects and runs with high motion. The to-be-censored frames (outliers) were identified by three stages. First, volumes with FD > 0.2 mm or DVARS > 75 were marked as outliers. Second, one frame before and two frames after these volumes were also flagged as outliers. Finally, remaining segments of data that lasted fewer than five contiguous volumes were censored. All censored frames marked in these three steps were discarded when computing global signal beta maps. BOLD runs with more than half of the frames flagged as censored were removed, resulting in 1030 subjects after motion censoring. Note that the FD threshold we picked is more conservative than that used in previous literature^11,46,60,61^. The DVARS was selected to achieve a similar number of censored frames as flagged by the FD threshold. In addition, two subjects did not have adequate family structure information (as used in the permutation testing procedure described below), leaving 1028 subjects for the final CCA.

### Delineation of global signal beta maps

The procedure to generate global signal beta maps is shown in Fig. 1. For each subject and each run, the global signal was calculated by averaging the timeseries across all cortical vertices in grey matter. Then for each run, the global signal and a vector of ones were regressed together (i.e. with bias term/intercept) from the fMRI signal for each vertex in the fs_LR space using ordinary least squares. To exclude the effects of high motion, censored frames were ignored when univariate linear regression was performed to regress the overall GS against each voxel’s BOLD activity fluctuations^34,62^. The ensuing regression (beta) coefficient corresponds to the differences in the global signal for each vertex. This constituted the global signal beta map for each scan. Since global signal beta maps were computed from multiple scans for each subject, we averaged the maps from multiple scans for each subject. The averaged beta map captures the overall relationship between the global signal and the BOLD signal at each vertex of the examined subject. The average global signal beta map for each subject was used for further analyses.

It is worth noting that there are multiple ways of computing the GS in the literature. While we computed the GS using just the cortex^34,63^, one can also compute the GS using the whole brain, or all gray matter voxels^51^. However, all definitions of GS lead to very similar results. For example, in the current data, the average correlation between GS computed from all gray matter locations (i.e., grayordinates) and the GS computed from just the cortical surface was 0.95. Thus, it is unlikely that alternate definitions of the GS would significantly alter our results.

### Canonical correlation analysis

To interrogate the relationship between the GS and behavior, we conducted a CCA. CCA is a natural choice of method because this machine-learning algorithm computes linear combinations of the original variables for each of two multivariate datasets that, together, maximize the linear correspondence between both variable sets. Each ensuing canonical mode, reflecting a pair of canonical variates, is indicated by a linear weighting of behavioral measures and a linear weighting of cortical vertices that are maximally correlated with each other. The strength of association (*r*) between the two variable sets was tested for robustness and statistical significance using a statistical null-hypothesis testing permutation framework^24^.

### Behavioral data exclusion and pre-processing

Several behavioral measures contain large amounts of missing observations, have heavily skewed distributions, and/or may represent potential confounds. To ensure these variables did not adversely affect our analysis, we followed a similar data-exclusion and pre-processing procedure as that described in^24^:

1. Nine measures were defined as confounds, and regressed out from the data (including each of the nine measures squared). These measures included *acquisition Reconstruction Software Version*, *Head Motion (Mean DVARS and Mean FD)*, *Weight*, *Height*, *Blood Pressure - Systolic*, *Blood Pressure - Diastolic*, *Hemoglobin A1c*, *Cube-Root of Total Brain Volume and Intracranial Volume*.
2. Behavioral measures were excluded from the CCA if they did not meet the following criteria: number of missing observations exceeds 500, the measure contained extreme outliers (defined as 100 standard deviations above the median), the standard deviation of the measure was greater than zero, or the size of the largest equal-values-group exceeds 95% of the observations.
3. Behavioral measures that were redundant or not of interest for the current analysis included (for variable descriptions see https://wiki.humanconnectome.org/display/PublicData/HCP+Data+Dictionary+Public-+Updated+for+the+1200+Subject+Release): *Age*, *Gender*, *Race*, *Ethnicity*, *Employment*, *Income*, *In School?*, *Missouri Born?*, *BMI*, *BMI Category*, *BMI Category Heaviest*, *Blood Drawn?*, *Hematocrit 1*, *Hematocrit 2*, *TestRetest Interval*, *Thyroid Hormone*, all *Hypothyroid* and *Menstrual* measures, *PMAT24_SI*, *PMAT_RTCR*, all *DDisc* measures (excluding the AUC measures), all *SCPT* measures (excluding *SCPT_SEN* and *SCPT_SPEC*), *IWRD_TOT*, *IWRD_RTC*, *ER40_CRT*, *Mars_Errs*, all *Endurance*, *GaitSpeed*, *Dexterity*, and *Strength* measures, and *Eye Color Vision*.

In total, 143 behavioral measures were excluded (including confounds). Following removal of these variables, all categorical variables were dummy-coded, and all variables were then z-score normalized. This resulted in a battery of 177 standardized behavioral measures for the CCA analysis. See Supplementary Fig. 2 for analyses without behavioral data exclusion.

### Data reduction

To avoid overfitting in the CCA analysis, and to contend with the high dimensionality of the datasets, we conducted a principal component analysis (PCA) individually on the pre-processed behavioral measures and on the global signal beta maps (z-score normalized), analogous to previous work^24^. Since we obtained a global signal beta map for each subject, we treated every subject as an observation and each vertex as a feature dimension in PCA. For example, the first principal component of global signal beta maps captured the most inter-subject variance in the global signal spatial topography. 100 principal components were derived from each of the behavioral and global signal beta maps. As in^24^, the covariance matrix of the behavioral measures was projected onto the nearest positive-definite covariance matrix, which avoided any imputation of missing observations.

A CCA was then conducted on the 100 behavioral and 100 global signal principal component scores. CCA estimates canonical variate pairs, or linear combinations of the behavioral with its links to a linear combination of global signal principal components, that are maximally correlated. Canonical variate pairs are organized in terms of decreasing magnitude, meaning the first canonical variate pair explains the largest amount of variation in the data, followed by the mode with second-largest explained variance, and so forth. The maximal number of canonical components (N = 100) was estimated. The CCA output of interest was the overall strength of the correlation between each canonical variate pair; the pattern of canonical weights for the original behavioral measures, on the one hand, and cortical vertices on their canonical variate, on the other hand. The canonical weights for each measure and cortical vertex were computed as the correlation between the canonical variate scores and the original behavioral measures and GS beta values across subjects.

### Statistical significance testing of canonical variate correlations (r)

To test for the statistical significance of the correlation strength (*r*) for all discovered brain-behavior associations (e.g. canonical variate pairs), we carried out the null permutation testing framework used in^24^. This flexible testing scheme aimed at rejecting the null hypothesis that the obtained pairs of canonical variates are likely due to random noise linking our brain and behavior data. The permutation test proceeded as follows: (1) subjects (i.e. rows) behavioral principal component scores were randomly re-ordered, respecting family structure^64^, to intentionally break the dependence structure between the two variable sets, (2) the CCA analysis was re-run between the original GS principal component scores and the re-ordered behavioral principal component scores, and (3) the first canonical variate pair’s correlation coefficient (the maximum possible correlation coefficient) was placed into a null distribution of correlation coefficient values supposing a lack of brain-behavior association, (4) this process was repeated 10,000 times, and (5) the original canonical variate pairs were declared statistically significant if their associated correlation coefficients exceeded the 99.9% percentile (i.e., *p* < 0.001 of the null distribution defined above). As noted in *Results*, only the first canonical variate pair was statistically significant (*p* < 0.001).

## Supplementary information

Supplementary Information

## Data Availability

The MRI and behavioral datasets used in this study are available to the public from the Human Connectome Project (S1200 release; https://www.humanconnectome.org/study/hcp-young-adult/document/1200-subjects-data-release).

## Code Availability

All code used to analyze data and create figures for the current study are available with no restrictions. Customized Matlab code can be accessed here: https://github.com/tsb46/Global-Signal-CCA-Analysis.
