# Supplementary material for: Topography and behavioral relevance of the global signal in the human brain
Source: Sci Rep. 2019 Oct 3;9:14286. doi: 10.1038/s41598-019-50750-8 (PMC6776616; doi:10.1038/s41598-019-50750-8)
Supplement: Supplementary file 1 — Supplementary Information [file 41598_2019_50750_MOESM1_ESM.pdf]

Topography and behavioral relevance of the global signal in the human brain

Supplementary Materials

Jingwei Li[1†], Taylor Bolt[2†], Danilo Bzdok[3,4,5], Jason S. Nomi[6], B. T. Thomas Yeo[1],
R. Nathan Spreng[7,8*], Lucina Q. Uddin[6,9*]

[1] ECE, CIRC, N.1 & MNP, National University of Singapore, Singapore
[2] Department of Biomedical Engineering, Emory University, Atlanta, GA, USA
[3] Department of Psychiatry, Psychotherapy and Psychosomatics, Aachen University, Aachen, Germany; [4] JARA, Translational Brain Medicine, Aachen, Germany; [5] Parietal Team, INRIA, Neurospin, bat 145, CEA Saclay, 91191 Gif-sur-Yvette, France
[6] Department of Psychology, University of Miami, Coral Gables, FL, USA
[7] Laboratory of Brain and Cognition, Montreal Neurological Institute, Department of Neurology and Neurosurgery, McGill University, Montreal, QC, Canada
[8] Departments of Psychiatry and Psychology, McGill University, Montreal, QC, Canada
[9] Neuroscience Program, University of Miami Miller School of Medicine, Miami, FL, USA

[†]These two authors contributed equally
*Correspondence should be addressed to:

R. Nathan Spreng
Montreal Neurological Institute
Department of Neurology & Neurosurgery
McGill University
3801 University St.
Montreal, QC, H3A 2B4, Canada
Email: nathan.spreng@gmail.com
Phone: (514) 398-7268

Or

Lucina Q. Uddin
University of Miami
Department of Psychology
P.O. Box 248185-0751
Coral Gables, FL 33124, USA
Email: l.uddin@miami.edu
Phone: (305) 284-3265

Supplementary Figures

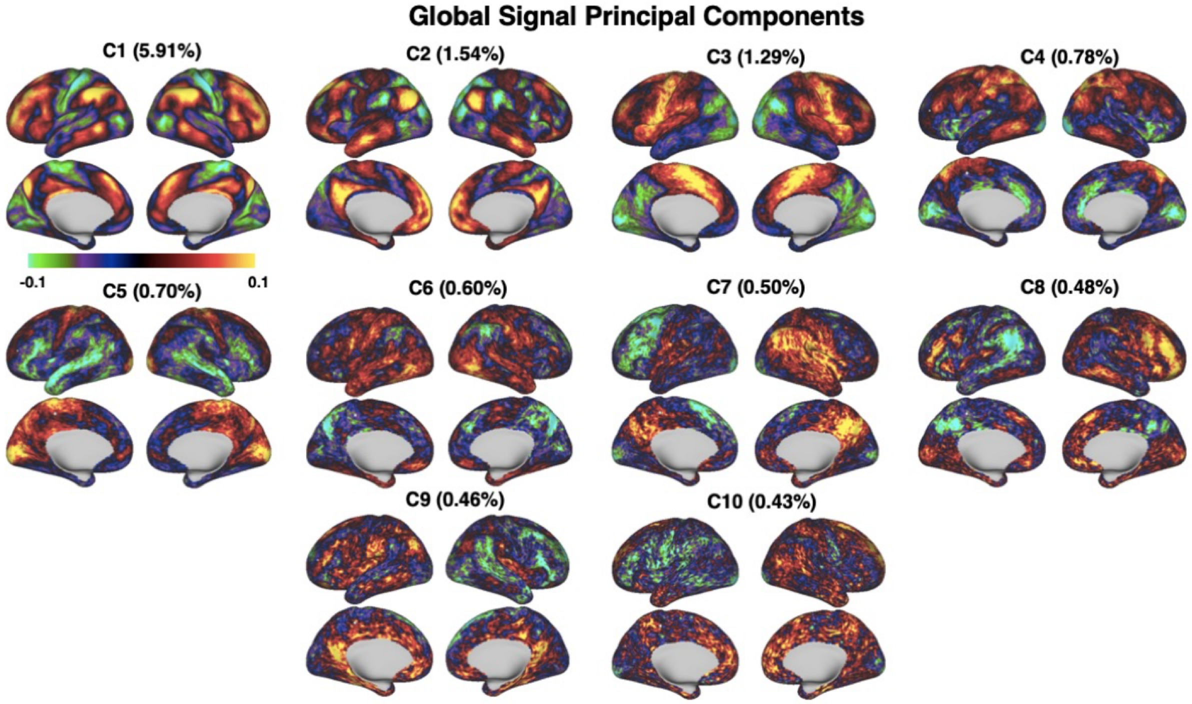

Supplementary Figure 1. First Ten Principal Components of the Global Signal Beta Estimates. Displayed are the first ten principal components computed across subjects from their global signal beta estimates.

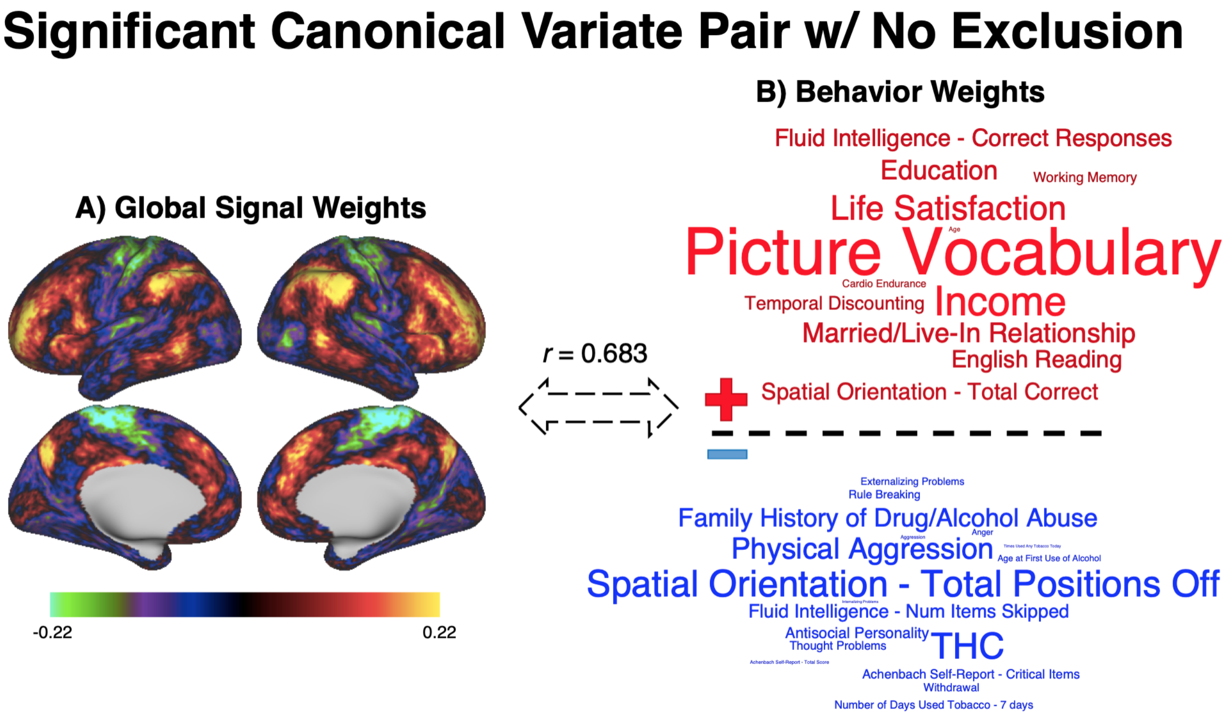

Supplementary Figure 2. Statistically Significant Canonical Variate Pair With No Variables Excluded. Results of CCA analysis with no behavioral variables excluded. **A)** CCA weights of each vertex on the first canonical variate pair. **B)** Top 20% positive (blue) and negative (red) behavioral variable CCA weights displayed in a word cloud. The size of the text in the word cloud is proportional to the absolute value of that variable's CCA weight.
